# Evaluation of the Safety and Efficacy of Xiao Yao San as a Treatment for Anxiety: A Systematic Review and Meta-Analysis

**DOI:** 10.1155/2022/1319592

**Published:** 2022-04-06

**Authors:** Jin Lin, Yue Ji, Jinhua Si, Guanran Wang, Xinju Li, Li Shen

**Affiliations:** ^1^Department of Psychosomatic Medicine, First Teaching Hospital of Tianjin University of Traditional Chinese Medicine, Tianjin 300381, China; ^2^National Clinical Research Center for Chinese Medicine Acupuncture and Moxibustion, Tianjin 300381, China; ^3^Graduate School, Tianjin University of Traditional Chinese Medicine, Tianjin, China; ^4^Library, Tianjin University of Traditional Chinese Medicine, Tianjin, China; ^5^Tianjin University of Traditional Chinese Medicine, Tianjin 301617, China

## Abstract

**Objective:**

Xiaoyao San (XYS) is a medicinal preparation that is commonly employed in China for the treatment of anxiety disorders (AD). Despite suggestions that it may offer certain advantages in this context, there are no reliable evidence-based studies regarding its efficacy at present. The present study was developed to gauge the efficacy and safety of XYS for the treatment of AD in a systematic manner.

**Methods:**

PubMed, the Cochrane Library, EMBASE, Web of Science, China National Knowledge Infrastructure (CNKI), Wanfang database, Weipu database, and China Biomedical Documentation Service System (CBM) databases were systematically searched for all randomized control trials (RCTs) evaluating the use of XYS for the treatment of AD published as of November 2021. Two investigators independently screened all studies, extracted data, and assessed the risk of bias for included studies using RevMan5.3.

**Results:**

In total, 9 RCTs incorporating 809 patients were included in the present meta-analysis, of which 3 compared oral XYS to anxiolytic treatment and 6 compared oral XYS + anxiolytics to anxiolytic treatment alone. The resultant meta-analysis revealed that XYS alone or in combination with anxiolytic treatment was associated with better improvements in anxiety-related symptoms and reduced adverse drug-related reactions as compared to anxiolytic treatment alone.

**Conclusion:**

The available evidence suggests that oral XYS alone or in combination with anxiolytic agents is more effective and safer than anxiolytic treatment alone when used for the treatment of AD. However, owing to the limited number and quality of the studies included in this analysis, further high-quality research will be essential to validate these results.

## 1. Introduction

Anxiety is an adverse emotional state in which individuals experience unease or nervousness that can be difficult to cope with. Anxiety disorders (ADs) are a group of psychological disorders characterized primarily by anxiety [1]. AD is associated with a lifetime prevalence of 13.6%–28.8% and an annual incidence rate of 5.6%–19.3%. An estimated 1% of all disability-adjusted life years are estimated to be lost due to anxiety-related factors such as panic attacks, obsessive-compulsive disorder, and posttraumatic stress disorder [2]. Rising levels of social pressure are resulting in rising annual AD incidence rates [3]. First-line pharmacological treatments for AD include a range of anxiolytics such as selective serotonin reuptake inhibitors (SSRIs), benzodiazepines, serotonin-norepinephrine reuptake inhibitors (SNRIs), noradrenergic and specific serotonergic antidepressants (NaSSAs), tricyclic antidepressants (TCAs), and azapirones [4]. While clinical trials have confirmed that these agents can very effectively treat AD [5–8], they are associated with adverse drug reactions and withdrawal symptoms that can make them undesirable for some patients [9], underscoring the need for the development of alternative safe and effective treatments. Traditional Chinese medicine (TCM) approaches offer advantages including excellent safety profiles and multitarget multipathway mechanisms of action, providing effective complementary and alternative treatments for AD [10].

The etiological basis for psychological illnesses and associated treatment methods is highly varied [11]. In TCM theory, the pathogenesis of AD is primarily believed to be associated with the stagnation of the liver and qi together with the dysfunction of the five internal organs, with excess and deficiency also contributing to this condition [12]. Xiaoyao San (XYS) is a TCM preparation consisting of Chai Hu (*Bupleurum*), Dang Gui (*Angelica*), Bai Shao (white peony), Bai Zhu (*Atractylodes*), Fu Ling (Poria), Sheng Jiang (ginger), Bo He (peppermint), and Zhi Gan Cao (roasted licorice). XYS is widely used for the treatment of anxiety and has been reported to relieve depression, soothe the liver, and strengthen the blood and spleen [13]. A number of recent studies have evaluated the efficacy of XYS as a treatment for AD, and multiple randomized controlled trials (RCTs) have found that it is superior to control treatments in terms of improved efficacy, shorter duration of treatment, and lower rates of related adverse drug reactions. Several basic research studies have also suggested that XYS exhibits anxiolytic activity when used to treat AD. For example, Sun et al. employed XYS for the treatment of chronic stress injury model rats and assessed hippocampal *Gabra4* gene expression in these animals, revealing that such treatment was sufficient to downregulate *Gabra4* and to thereby alleviate chronic stress-related damage via soothing the liver and alleviating anxiety [14]. However, different studies have employed different XYS treatment strategies and study designs, making it challenging to draw reliable conclusions regarding the utility of this TCM preparation. The present systematic review was thus constructed to explore the safety and efficacy of XYS as a treatment for AD in an effort to provide an evidence-based foundation for future research and clinical treatment efforts.

## 2. Methods

This study was conducted as per the Preferred Reporting Items for Systematic Reviews and Meta-analysis (PRISMA) guidelines [14] and is registered in PROSPERO (registration number: CRD42021285024).

### 2.1. Search Strategy

The PubMed, the Cochrane Library, EMBASE, Web of Science, China National Knowledge Infrastructure (CNKI), Wanfang database, Weipu database, and China Biomedical Documentation Service System (CBM) databases were searched for all relevant studies published as of November 2021 using a combination of subject words and free words. Retrieval words included anxiety disorder, anxiety state, Xiaoyao San, and Xiaoyao Pill (Appendix 1). All literature searches were independently performed by two investigators (Jin Lin and Yue Ji), with disagreements being resolved through discussion with a third investigator (Jinhua Si).

### 2.2. Inclusion and Exclusion Criteria

#### 2.2.1. Inclusion Criteria

1. Study type: RCTs exploring the use of XYS for the treatment of AD.
2. Diagnostic criteria: patients were diagnosed with AD as per the criteria included in the “Chinese Classification and Diagnostic Criteria for Mental Disorders” [15], with TCM diagnostic criteria being made in reference to the “Criteria for Diagnosis and Efficacy of TCM Diseases.”
3. Intervention measures: patients in the treatment group were treated with oral XYS either alone or in combination with other anxiolytic drugs, while patients in the control group were treated with the same anxiolytic agents used in the treatment group. XYS oral preparations eligible for inclusion in this analysis included XYS granules, XYS soup (XYS with the addition or removal of up to three herbs based on patient symptoms), and other dosage strategies.
4. Outcome indicators: the primary outcome indicators for this study included: (1) the total efficacy rate as a means of gauging reductions in Hamilton anxiety scale (HAM-A) scores. For this endpoint, anxiety reduction rates were scored as follows: a reduction rate >75% was considered to be indicative of recovery, while a reduction rate >50% was considered a marked effect, a reduction rate ≥25% was considered effective, and a reduction rate <25% was considered ineffective. The total efficacy rate was calculated as follows: total efficacy = (recovery number + marked effect number + effective number)/total number ^*∗*^  100%. (2) HAM-A scores.

Secondary outcome indicators included: (1) Self-Rating Anxiety Scale (SAS) values; (2) Treatment Emergent Symptom Scale (TESS) values; (3) Traditional Chinese Medicine Symptom Observation Scale scores; and (4) adverse reaction rates.

#### 2.2.2. Exclusion Criteria

1. Duplicate studies or republished datasets were excluded, with only the most complete and highest quality study being included in the pooled analysis
2. Studies with incomplete data or obvious errors that could not be corrected by contacting the corresponding author were excluded
3. Studies that did not report observation outcome indicators were excluded
4. The use of other TCM treatment techniques (such as acupuncture and massage) during the treatment process led to study exclusion regardless of whether it was in the treatment group or the control group

### 2.3. Study Selection and Data Extraction

Study screening was independently performed by two investigators with reference to the above inclusion and exclusion criteria, with disagreements being resolved through discussion and consensus or consultation with a third investigator. The data extracted from included studies included: (i) basic information including title, first author, publication year, numbers of patients per group, and baseline patient characteristics; (ii) interventional measures and treatment courses for the treatment and control groups; (iii) outcome indicators; and (iv) elements necessary for risk of bias assessments.

### 2.4. Risk of Bias Analysis

The risk of bias for included studies was independently quantified by two investigators, with disagreements being resolved through discussion with a third investigator. The risk of bias was measured with the RCT bias risk assessment tool from the Cochrane Manual 5.1.0 [16].

### 2.5. Statistical Analysis

RevMan5.3 was used to conduct the present meta-analysis. When continuous data were measured using the same measurement tools and units, they were analyzed based on weighted mean difference (WMD) values, whereas they were otherwise analyzed using standard mean difference (SMD) values. Dichotomous variables were analyzed using relative risk (RR) values and 95% confidence intervals (95% CIs). The chi-squared test was used to detect heterogeneity among studies with the I^2^ statistic. When no significant heterogeneity was detected (*P* > 0.10, *I*^2^ < 50%), results were analyzed with a fixed-effects model. When significant heterogeneity was detected (*P* > 0.10, *I*^2^ ≥ 50%), subgroup or sensitivity analyses were used to explore potential sources of heterogeneity. When clear sources of clinical or methodological heterogeneity had been removed, a pooled meta-analysis was conducted using a random-effects model. The influence of individual studies on pooled results was assessed through sensitivity analyses, with individual studies being removed from the overall analysis to look for sources of heterogeneity. For primary outcome indicators, when 10 or more studies were available, publication bias was detected via visual inspection of funnel plots and through Egger's test and Begg's test.

## 3. Results

### 3.1. Study Characteristics

**Figure 1:**
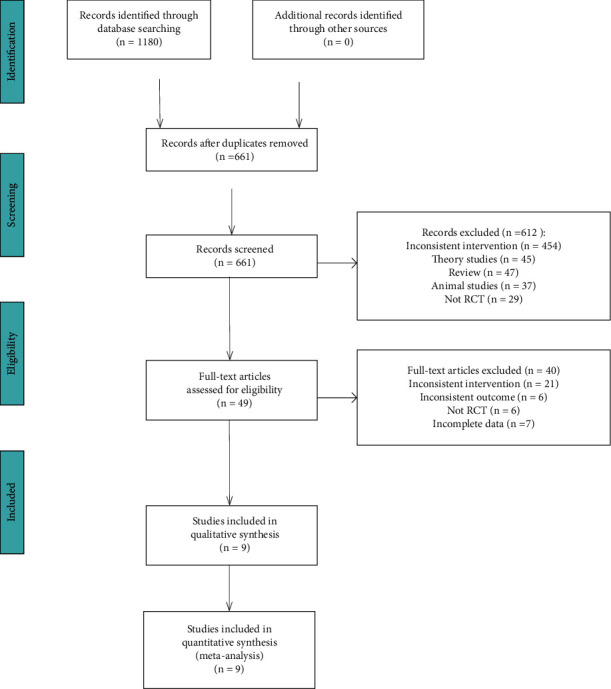
Flow diagram.

In total, the initial search strategy retrieved 1180 potentially relevant studies, of which 661 remained following the removal of duplicates. Of these, 612 were excluded following preliminary abstract and title reviews, while 40 were excluded following full-text review. The remaining 9 studies were included in the final analysis. The overall screening process is detailed in Figure 1.

The key characteristics of the included studies are listed in Table 1. The treatment group consists of patients treated with XYS alone or in combination with other antianxiety drugs, while the control group consists of patients treated with antianxiety drugs only. Interventional approaches for the included studies are listed in Table 2.

### 3.2. Risk of Bias Analysis

**Figure 2:**
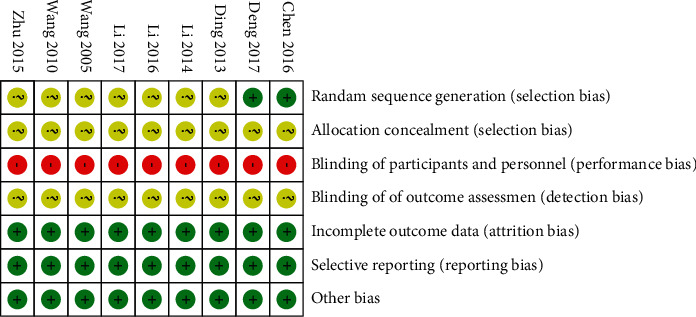
Risk of bias summary.

An RCT approach was employed to assess the quality of the 9 studies included in the present meta-analysis as per the Cochrane Manual 5.1.0. Just two studies employed appropriate random sequence generation methods [17, 18], while no studies mentioned the use of appropriate blinding techniques [17–25]. All studies exhibited an unclear risk of bias with respect to allocation concealment and outcome assessment blinding [17–25]. Moreover, all studies exhibited a low risk of bias with respect to selective reporting, incomplete outcome indicators, and other forms of bias [17–25]. The results of these analyses are listed in Figure 2.

### 3.3. Meta-Analysis Results

Of the 9 included studies, 3 compared oral XYS alone to anxiolytics, while 6 compared oral XYS + anxiolytics with anxiolytics.

#### 3.3.1. Oral XYS Alone vs. Anxiolytics

**Figure 3:**
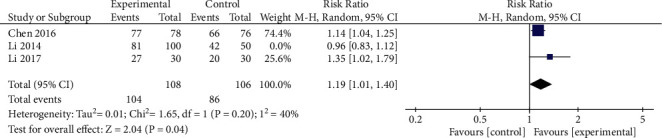
Meta-analysis of total efficacy rates for oral XYS alone vs. anxiolytics.

*(1) Total Efficacy Rates*. Total efficacy rates were reported by 3 of the included RCTs, and significant heterogeneity was detected among the results of these analyses (*P*=0.07, *I*^2^ = 63%). When studies were iteratively omitted from this analysis, the exclusion of the study conducted by Li et al. eliminated this heterogeneity (*P*=0.20, *I*^2^ = 40%, Figure 3), indicating that this study was a source of substantial heterogeneity. The pooled data were then analyzed with a random-effects model, the efficacy of oral XYS was found to be significantly superior to that of oral anxiolytic treatment ([RR = 1.19, 95% CI: 1.01, 1.40, *P*=0.04]).

*(2) HAM-A Scores*. HAM-A scores were only reported in a single trial, thus precluding the performance of a meta-analysis. Descriptive analysis of these results indicated that scores in the treatment group were significantly reduced relative to the control group ([MD = −6.52, 95%CI: −7.45, −5.59]), *P* < 0.00001]).

*(3) TCM Syndrome Scale*. The TCM Syndrome Scale was only reported in a single RCT, thus precluding the performance of a pooled meta-analysis. Descriptive analysis indicated that the scores for the treatment group were significantly lower than those for the control group ([MD = −3.30, 95%CI: −4.16, −2.44]), *P* < 0.00001]).

**Figure 4:**
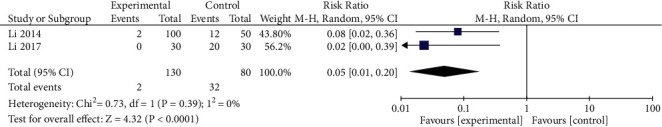
Meta-analysis of adverse event rates when comparing oral XYS alone vs. anxiolytics.

*(4) Adverse Event Rates*. Adverse event rates were reported by 2 RCTs, with no significant heterogeneity being observed for the pooled results (*P*=0.39, *I*^2^ = 0%). Data were thus analyzed using a fixed-effects model, revealing that adverse event rates were significantly lower in the treatment group relative to the control group ([RR = 0.05, 95% CI: 0.01, 0.20, *P* < 0.0001]; Figure 4).

#### 3.3.2. Oral XYS + Anxiolytics *vs.* Anxiolytics Alone

**Figure 5:**
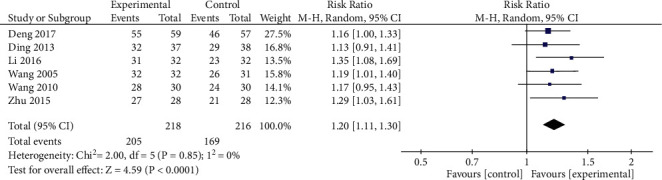
Meta-analysis of total efficacy rates when comparing oral XYS + anxiolytics vs. anxiolytics alone.

**Figure 6:**
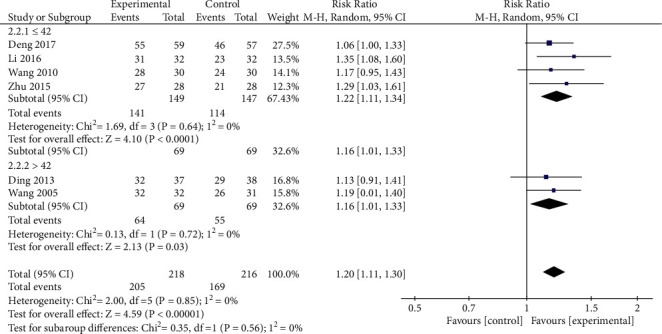
Subgroup analysis comparing total efficacy rates for oral XYS + anxiolytics to those for anxiolytics alone.

*(1) Total Efficacy Rates*. In total, 6 RCTs reported total efficacy rates, with no significant heterogeneity among these studies (*P*=0.85, *I*^2^ = 0%). Data were analyzed with fixed-effects models, revealing significantly better total efficacy rates in the treatment group relative to the control group ([RR = 1.20, 95% CI: 1.11, 1.30, *P* < 0.00001], Figure 5). As there was substantial variability with respect to treatment duration among these different studies, we additionally conducted a subgroup analysis based on differences in treatment course by separating patients into those treated for ≤42 days and >42 days. Heterogeneity analyses revealed no significant heterogeneity for the ≤42 day (*P*=0.64, *I*^2^ = 0%), or >42 day (*P*=0.72, *I*^2^ = 0%) treatment groups (Figure 6), with fixed-effects models thus being used for pooled analysis. In both the ≤42 day and >42 day treatment groups, oral XYS + anxiolytic treatment was associated with better efficacy than that observed for oral anxiolytics alone (≤42 days [RR = 1.22, 95% CI: 1.11, 1.34, *P* < 0.0001]; >42 days [RR = 1.16, 95% CI: 1.01,1.33, *P*=0.03]).

**Figure 7:**
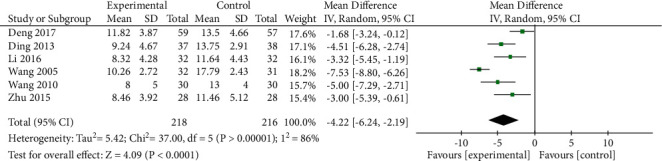
Meta-analysis comparing HAM-A scores for oral XYS + anxiolytics to those for Anxiolytic treatment alone.

**Figure 8:**
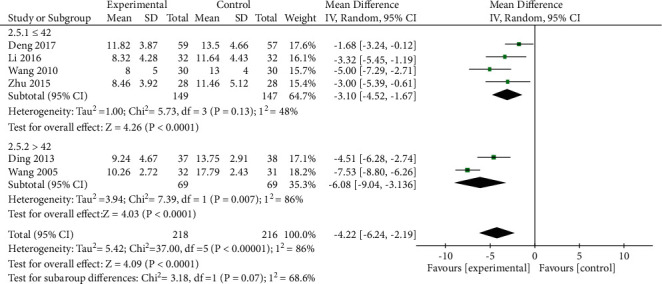
Subgroup analysis comparing HAM-A scores for oral XYS + anxiolytic treatment to those for Anxiolytics alone.

*(2) HAM-A Scores*. HAM-A scores were reported in 6 studies, and significant heterogeneity was detected among these studies (*P* < 0.00001, *I*^2^ = 86%). However, the confidence interval for the forest plot was to the left of the invalid line, indicating that such heterogeneity had no impact on the overall results. Pooled data were thus analyzed with a random-effects model. Pooled analysis indicated that HAM-A scores were significantly lower in the treatment group relative to the control group ([MD = −4.22, 95% CI: −6.24, −2.19, *P* < 0.0001]; Figure 7). Next, subgroup analyses were conducted based on treatment course in order to identify sources of heterogeneity. Significant heterogeneity was detected in the >42 day treatment subgroup (*P*=0.007, *I*^2^ = 86%), while no significant heterogeneity was detected in the ≤42 day treatment subgroup (*P*=0.13, *I*^2^ = 48%) (Figure 8). Next, heterogeneity was assessed for subgroups of patients with pretreatment HAM-A scores >29 points and pretreatment HAM-A scores >14 and ≤29 points. However, significant heterogeneity was still detected in this pooled analysis, indicating that HAM-A scores or numbers of treatment days were not the sources of heterogeneity in the pooled result analysis. Sensitivity analysis additionally failed to significantly decrease or increase heterogeneity or effect size. Given that the overall heterogeneity was relatively low and that no individual study biased these results, these results suggest that oral XYS + anxiolytic treatment can achieve superior efficacy to anxiolytic treatment alone as a means of lowering AD patient HAM-A scores.

*(3) SAS*. SAS scores were reported in just 1 RCT. As a meta-analysis could not be performed, descriptive analysis was instead conducted, revealing significantly lower SAS scores in the treatment group relative to the control group ([MD = −4.56, 95% CI: −7.19, −1.93]), *P*=0.0007]).

**Figure 9:**
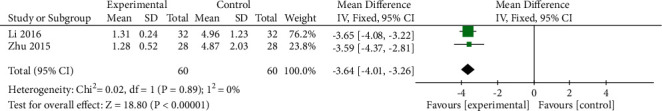
Meta-analysis comparing TESS scores for Oral XYS + Anxiolytic treatment to those for Anxiolytics alone.

*(4) TESS*. TESS scores were reported in 2 RCTs. No heterogeneity was detected when analyzing these data (*P*=0.89, *I*^2^ = 0%), and results were thus analyzed with a fixed-effects model. Pooled analysis indicated that TESS scores in the treatment group were significantly lower than those in the control group ([MD = 3.64, 95% CI: −4.01, −3.26, *P* < 0.00001]; Figure 9).

**Figure 10:**
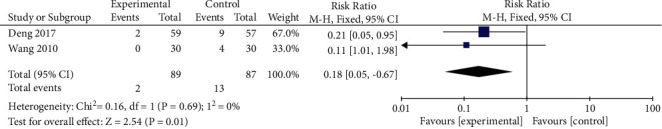
Meta-analysis comparing adverse event rates for oral XYS + anxiolytic treatment to those for anxiolytics alone.

*(5) Adverse Event Rates*. Adverse event rates were reported by two of the included RCTs. No heterogeneity was detected when evaluating these studies (*P*=0.69, *I*^2^ = 0%), and results were thus analyzed with a fixed-effects model. The pooled meta-analysis indicated that adverse event rates in the treatment group were significantly lower than those in the control group ([RR = 0.18, 95% CI: 0.05, 0.67, *P*=0.01 < 0.05]; Figure 10).

## 4. Discussion

Here, we conducted a pooled meta-analysis of 9 RCTs in which XYS was employed for the treatment of AD. The results of these analyses indicated that XYS treatment, either alone or in combination with anxiolytic agents, was superior to anxiolytic treatment alone with respect to total efficacy rates. Subgroup analysis further indicated that this effect remained evident irrespective of treatment duration. Moreover, we found that HAM-A scores, which are commonly used to assess anxiety symptoms, improved more significantly for patients treated with XYS than for patients treated with anxiolytics, irrespective of treatment duration or pretreatment HAM-A scores. SAS scores are used to assess anxiety severity, and while only one study assessed the scores in patients undergoing oral XYS + anxiolytic treatment, descriptive analysis indicated that such treatment was superior to anxiolytic treatment alone. The TCM Syndrome Scale is used to evaluate patient discomfort symptoms. As relatively few studies included this scale, a descriptive analysis was instead conducted, revealing that XYS treatment alone was superior to anxiolytic treatment. With respect to adverse event rates, fewer adverse reactions were reported in the XYS and XYS + anxiolytic groups compared to anxiolytic treatment alone. A meta-analysis of the results comparing the effects of oral XYS + anxiolytic treatment to anxiolytic treatment revealed scores were significantly lower in the treatment group relative to the control group. These data suggest that oral XYS is thus safe and effective as a treatment for AD, reducing drug treatment-related adverse reactions.

Anxiety is a psychological condition in which individuals experience episodes of distress and unease that can adversely affect their social function [26]. The prolonged alertness experienced by those with anxiety can increase the risk of cardiovascular disease and cerebrovascular disease [27]. Chinese medicinal approaches draw from thousands of years of experience, offering many advantages as treatments for psychological disorders [28].

XYS is among the most frequently utilized TCM preparations for the treatment of psychological illnesses, and it has been shown to exhibit psychotropic and anxiolytic activity in animal model studies [29]. Several reports have suggested that XYS may exert its anxiolytic activity in part through modulation of the intestinal microflora, increasing the relative abundance of anaerobic bacteria within the intestines and the associated production of intestinal-derived short-chain fatty acids (SCFAs). These changes can prevent bacterial migration and reduce peripheral inflammation, with neuroinflammation ultimately being alleviated through changes in both central and peripheral immunity, thereby mediating an antianxiety effect [30]. *Bupleurum* is the key drug in many spiritual prescriptions, and several pharmacological studies have suggested that it can relieve neuronal apoptosis and associated neuroinflammation [31], increasing concentrations of nerve growth factors and brain-derived neurotrophic factor [32].

This study is subject to several limitations. For one, many of the included studies did not specify the allocation concealment or blinding approaches employed, and the results may thus be susceptible to measurement bias and selection basis. In addition, this study did not assess the degree of anxiety, education level, previous life experience, or concomitant diseases in the included patients. These factors will affect the treatment of anxiety, and their omission may lead to heterogeneity among study results. In addition, all the studies were from China and may thus not be generalizable. There were also differences in the dosage and composition of XYS used in these different studies, potentially influencing pooled analysis results.

## 5. Conclusions

Current research suggests that XYS treatment can effectively alleviate AD patients' anxiety symptoms while reducing rates of adverse drug reactions as compared to anxiolytic treatment. The overall efficacy of XYS alone or in combination with anxiolytic agents was no less than that of anxiolytic agents alone in our pooled analysis. However, to validate these results, additional large-scale multicenter high-quality clinical trials will be essential, thereby providing a foundation for future patient treatment.

## Figures and Tables

**Table 1:** Included study characteristics.

Study cohort	No (T/C)	Gender		Age		Course (day)	Outcome
		T	C	T	C		
Chen and Lj [17]	81/78	40/41	40/38	43.65 ± 8.01	43.93 ± 10.56	42	①②⑤
Deng [18]	59/57	26/33	30/27	43.81 ± 10.23	44.53 ± 11.65	28	①②⑥
Ding [19]	37/38	—	—	29.46 ± 6.82	27.62 ± 4.24	56	①②③
Li [20]	100/50	—	—	—	—	56	①⑥
Li [20]	32/32	15/17	14/18	41.6 ± 7.8	42.1 ± 7.2	42	①②⑥
Li [21]	30/30	22/8	19/11	43–79	41–80	28	①⑥
Wang [22]	32/31	18/14	20/11	19–57	20–56	56	①②
Wang [23]	30/30	16/14	15/15	18–48	20–51	42	①②⑥
Zhu [24]	28/28	—	—	—	—	42	①②④

Note: outcome: ① efficiency; ② HAMA; ③ SAS; ④ TESS; ⑤ symptom rating scale of TCM; ⑥ adverse reaction rate; —: unclear.

**Table 2:** Intervention characteristics.

Study	Interventions of treatment group		Interventions of control group	Days
	XYS	Anxiolytics	Anxiolytics	
Chen and Lj [17]	Xiaoyao san decoction, 150 ml Bid	None	Flupentixol and melitracen tablets, 10.5 mg Bid	42
Deng [18]	Xiaoyao san granule, 5 g Bid	Paroxetine, 20–40 mg Qd	Paroxetine, 20–40 mg Qd	28
Ding [19]	Xiaoyao san decoction Bid	Buspirone, 5–10 mg Tid	Buspirone, 5–10 mg Tid	56
Li [20]	Xiaoyao san granule, 8 granule Tid	None	Alprazolam, 0.4–1.2 mg/day	56
Li [20]	Xiaoyao san decoction Bid	Buspirone, 10 mg Tid	Buspirone, 10 mg Tid	42
Li [20]	Xiaoyao san decoction, 200 ml Bid	None	Paroxetine, 20 mg Qd	28
Wang [22]	Xiaoyao san decoction, 150 ml Bid	Doxepin 25 mg tid + alprazolam 0.4–1.2 mg Bid + oryzanol 20 mg Tid	Doxepin 25 mg tid + alprazolam 0.4–1.2 mg bid + oryzanol 20 mg Tid	56
Wang [23]	Xiaoyao san granule, 9 g Tid	Flupentixol and melitracen tablets, 10.5 mg Bid	Flupentixol and melitracen tablets, 10.5 mg Bid	42
Zhu [24]	Xiaoyao san decoction, 150 ml Bid	Buspirone, 10 mg Tid	Buspirone, 10 mg Tid	42

## Data Availability

The data used to support the findings of this study are available from the corresponding author upon request.

## Abbreviations

AD: Anxiety disorders
SSRI: Selective serotonin reuptake inhibitor
SNRI: Serotonin norepinephrine reuptake inhibitor
NaSSA: Noradrenergic and specific serotonergic antidepressant
TCAs: Tricyclic antidepressants
TCM: Traditional Chinese medicine
XYS: Xiao yao san
RCTs: Randomized controlled trials
HAMA: Hamilton Anxiety Scale
SAS: Self-Rating Anxiety Scale
TESS: Treatment Emergent Symptom Scale
WMD: Weighted mean difference
SMD: Standard mean difference
RR: Relative risk.

## Supplementary Materials

Supplementary Materials Appendix 1. Search strategy in PubMed database. (Supplementary Materials). Appendix 2. The PRISMA checklist.
