# Supplementary material for: Evaluation of the Safety and Efficacy of Xiao Yao San as a Treatment for Anxiety: A Systematic Review and Meta-Analysis
Source: Evid Based Complement Alternat Med. 2022 Apr 6;2022:1319592. doi: 10.1155/2022/1319592 (PMC9007650; doi:10.1155/2022/1319592)
Supplement: Supplementary Materials — Appendix 1. Search strategy in PubMed database. (Supplementary Materials). Appendix 2. The PRISMA checklist. [file 1319592.f1.zip › 1319592.f1/polish report.pdf]

# EDITORIAL CERTIFICATE

This document certifies that the manuscript listed below was edited for grammar, punctuation, spelling, and overall style by one or more expert native English speaking editors with a PhD degree.

**Manuscript information**

**ID：MJ20698**

Editing date：2022.03.17

Title :Evaluation of the safety and efficacy of Xiao Yao San as a treatment for anxiety: a systematic review and meta-analysis

Authors :Jin Lin, Yue Ji, Jinhua Si, Guanran Wang,Xinju Li, Li Shen

Language writing before editing: ☐Very poor ☐Poor ☑Fair ☐Good ☐Very good ☐Excellent

Recommendation after language editing

☑Submitting to target journal directly
☐Submitting to target journal after minor revision
☐Re-editing required after major revision
☐Not suitable for publication

**Certificate by**

Sophiya.K

Editor in Chief
MJ Language Editing Services, Shenzhen, China

Disclaimer:Our service does not involve authenticity review or ethical review on the data (including images) presented in the manuscript. Neither the research content nor the author's intentions were altered in any way during the editing process. Documents receiving this certification should be English-ready for publication. The authors have the option to accept or reject our suggestions and changes in the edited document. However, we do not bear responsibility for revisions made to the document after our editing. If the manuscript is suspected of plagiarism, please contact the authors in time.

**MJ Language Editing Services**
Diwang Building, No. 5002 Shennan Road, Luohu District, Shenzhen, China
Tel:+086 0755 25100506
