# Supplementary material for: Evaluation of the Safety and Efficacy of Xiao Yao San as a Treatment for Anxiety: A Systematic Review and Meta-Analysis
Source: Evid Based Complement Alternat Med. 2022 Apr 6;2022:1319592. doi: 10.1155/2022/1319592 (PMC9007650; doi:10.1155/2022/1319592)
Supplement: Supplementary Materials — Appendix 1. Search strategy in PubMed database. (Supplementary Materials). Appendix 2. The PRISMA checklist. [file 1319592.f1.zip › 1319592.f1/PRISMA.docx]

| **Section and Topic** | **Item #** | **Checklist item** | **Location where item is reported** |
| --- | --- | --- | --- |
| **TITLE** | | |  |
| Title | 1 | Evidence-Based Complementary and Alternative Medicine Evaluation of the safety and efficacy of Xiao Yao San as a treatment for anxiety: a systematic review and meta-analysis | 1 |
| **ABSTRACT** | | |  |
| Abstract | 2 | Objective: Xiaoyao San (XYS) is a medicinal preparation that is commonly employed in China for the treatment of anxiety disorders (AD). Despite suggestions that it may offer certain advantages in this context, however, there are no reliable evidence-based studies regarding its efficacy at present. The present study was developed to gauge the efficacy and safety of XYS for the treatment of AD in a systematic manner. Methods: The PubMed, The Cochrane Library, EMBASE, Web of Science, China National Knowledge Infrastructure (CNKI), Wanfang database, Weipu database, and China Biomedical Documentation Service System (CBM) databases were systematically searched for all randomized control trials (RCTs) evaluating the use of XYS for the treatment of AD published as of November 2021. Two investigators independently screened all studies, extracted data, and assessed the risk of bias for included studies using RevMan5.3. Results: In total, 9 RCTs incorporating 809 patients were included in the present meta-analysis, of which 3 compared oral XYS to anxiolytic treatment and 6 compared oral XYS + anxiolytics to anxiolytic treatment alone. The resultant meta-analyses revealed that XYS alone or in combination with anxiolytic treatment was associated with better improvements in anxiety-related symptoms and reduced adverse drug-related reactions as compared to anxiolytic treatment alone. Conclusion: The available evidence suggests that oral XYS alone or in combination with anxiolytic agents is more effective and safer than anxiolytic treatment alone when used for the treatment of AD. However, owing to the limited number and quality of the studies included in this analysis, further high-quality research will be essential to validate these results. | 1 |
| **INTRODUCTION** | | |  |
| Rationale | 3 | Anxiety is an adverse emotional state in which individuals experience unease or nervousness that can be difficult to cope with. Anxiety disorders (Ads) are a group of psychological disorders characterized primarily by anxiety. AD is associated with a lifetime prevalence of 13.6%-28.8%, and an annual incidence rate is 5.6%-19.3%. An estimated 1% of all disability-adjusted life years are estimated to be lost due to anxiety-related factors such as panic attacks, obsessive-compulsive disorder, and post-traumatic stress disorder. Rising levels of social pressure are resulting in rising annual AD incidence rates. First-line pharmacological treatments for AD include a range of anxiolytics such as selective serotonin reuptake inhibitors (SSRIs), benzodiazepines, serotonin norepinephrine reuptake inhibitors (SNRIs), noradrenergic and specific serotonergic antidepressants (NaSSAs ), tricyclic antidepressants (TCAs), and azapirones. While clinical trials have confirmed that these agents can very effectively treat AD , they are associated with adverse drug reactions and withdrawal symptoms that can make them undesirable for some patients, underscoring the need for the development of alternative safe and effective treatments. Traditional Chinese medicine (TCM) approaches offer advantages including excellent safety profiles and multi-target multi-pathway mechanisms of action, providing effective complementary and alternative treatments for AD. | 2 |
| Objectives | 4 | The etiological basis for psychological illnesses and associated treatment methods are highly varied.In TCM theory, the pathogenesis of AD is primarily believed to be associated with the stagnation of the liver and qi together with the dysfunction of the five internal organs, with excess and deficiency also contributing to this condition. Xiaoyao San (XYS) is a TCM preparation consisting of Chai Hu (Bupleurum), Dang Gui (Angelica), Bai Shao (White Peony), Bai Zhu (Atractylodes), Fu Ling (Poria), Sheng Jiang (Ginger), Bo He (Peppermint), and Zhi Gan Cao (Roasted Licorice). XYS is widely used for the treatment of anxiety and has been reported to relieve depression, soothe the liver, and strengthen the blood and spleen. There have been many recent studies evaluating the efficacy of XYS as a treatment for AD, with multiple randomized controlled trials (RCTs) having found it to outperform control treatments with reducing the associated rates of adverse drug reactions. Several basic research studies have also suggested that XYS exhibits anxiolytic activity when used to treat AD. For example, Sun et al. employed XYS for the treatment of chronic stress injury model rats and assessed hippocampal Gabra4 gene expression in these animals, revealing that such treatment was sufficient to downregulate Gabra4 and to thereby alleviate chronic stress-related damage via soothing the liver and alleviating anxiety. However, different studies have employed different XYS treatment strategies and study designs, making it challenging to draw reliable conclusions regarding the utility of this TCM preparation. The present systematic review was thus constructed to explore the safety and efficacy of XYS as a treatment for AD in an effort to provide an evidence-based foundation for future research and clinical treatment efforts. | 2 |
| **METHODS** | | |  |
| Eligibility criteria | 5 | Inclusion and Exclusion CriteriaInclusion criteria  1. Study type: RCTs exploring the use of XYS for the treatment of AD.   (2) Diagnostic criteria: Patients were diagnosed with AD as per the criteria included in the "Chinese Classification and Diagnostic Criteria for Mental Disorders", with TCM diagnostic criteria being made in reference to the "Criteria for Diagnosis and Efficacy of TCM Diseases".  (3) Intervention measures: Patients in the treatment group were treated with oral XYS either alone or in combination with other anxiolytic drugs, while patients in the control group were treated with the same anxiolytic agents used in the treatment group. XYS oral preparations eligible for inclusion in this analysis included XYS granules, XYS soup (XYS with the addition or removal of up to three herbs based on patient symptoms), and other dosage strategies.  (4) Outcome indicators: The primary outcome indicators for this study included: ① The total efficacy rate as a means of gauging reductions in Hamilton anxiety scale (HAM-A) scores. For this endpoint, anxiety reduction rates were scored as follows: a reduction rate > 75% was considered to be indicative of recovery, while a reduction rate > 50% was considered a marked effect, a reduction rate ≥ 25% was considered effective, and a reduction rate < 25% was considered ineffective. The total efficacy rate was calculated as follows: Total efficacy = (nrecovery + nmarked effect + neffective)/ntotal * 100%. ② HAM-A scores.  Secondary outcome indicators included: ① Self-Rating Anxiety Scale (SAS) values; ② Treatment Emergent Symptom Scale (TESS) values; ③ Traditional Chinese Medicine Symptom Observation Scale scores; ④ Adverse reaction rates.  2.2.2. Exclusion criteria  (1) Duplicate studies or re-published datasets were excluded, with only the most complete and highest quality study being included in pooled analyses.  (2) Studies with incomplete data or obvious errors that could not be corrected by contacting the corresponding author were excluded.  (3) Studies that did not report observation outcome indicators were excluded.  (4) Analyses in which other TCM methods or interventional measures such as acupuncture, or similar techniques were used in the treatment group were excluded. | 3-4 |
| Information sources | 6 | The PubMed, The Cochrane Library, EMBASE, Web of Science, China National Knowledge Infrastructure (CNKI), Wanfang database, Weipu database, and China Biomedical Documentation Service System (CBM)databases were searched for all relevant studies published as of November 2021 using a combination of subject words and free words. Retrieval words included anxiety disorder, anxiety state, Xiaoyao San, and Xiaoyao Pill (see Appendix 1). All literature searches were independently performed by two investigators (Jin Lin and Yue Ji), with disagreements being resolved through discussion with a third investigator (Jinhua Si). | 3 |
| Search strategy | 7 | 1 "Anxiety"[MeSH Terms]/exp  2 “phobia*”[TI/AB] OR “panic”[TI/AB] OR “Angst”[TI/AB] OR“Nervousness”[TI/AB] OR “Hypervigilance”[TI/AB]OR “Anxiousness”[TI/AB] OR “Anxiet*”[TI/AB]OR “Castration Complex”[TI/AB]OR “Castration Complices”[TI/AB]OR "Catastrophizing*"[TI/AB]OR “Dental Fear*”[TI/AB]OR “Odontophobia*”[TI/AB]OR “Dental Phobia*”[TI/AB]OR “Exam Stress”[TI/AB]  3 1-2/OR  4"xiaoyao"[Supplementary Concept] /exp  5 "xiaoyao"[Text Word]) OR "xiao yao"[Text Word]  6 4-5/OR  7 "randomized controlled trial"[Publication Type] OR "controlled clinical trial" [Publication Type] OR "placebo"[TI/AB] OR "random*"[TI/AB] OR "clinical trial"[Publication Type] OR " trial"[TI/AB]  8 3 AND 6 AND 7 | Appendix 1 |
| Selection process | 8 | The PubMed, The Cochrane Library, EMBASE, Web of Science, China National Knowledge Infrastructure (CNKI), Wanfang database, Weipu database, and China Biomedical Documentation Service System (CBM)databases were searched for all relevant studies published as of November 2021 using a combination of subject words and free words. Retrieval words included anxiety disorder, anxiety state, Xiaoyao San, and Xiaoyao Pill (see Appendix 1). All literature searches were independently performed by two investigators (Jin Lin and Yue Ji), with disagreements being resolved through discussion with a third investigator (Jinhua Si). | 3 |
| Data collection process | 9 | The PubMed, The Cochrane Library, EMBASE, Web of Science, China National Knowledge Infrastructure (CNKI), Wanfang database, Weipu database, and China Biomedical Documentation Service System (CBM)databases were searched for all relevant studies published as of November 2021 using a combination of subject words and free words. Retrieval words included anxiety disorder, anxiety state, Xiaoyao San, and Xiaoyao Pill (see Appendix 1). All literature searches were independently performed by two investigators (Jin Lin and Yue Ji), with disagreements being resolved through discussion with a third investigator (Jinhua Si).. | 3 |
| Data items | 10a | Study screening was independently performed by two investigators with reference to the above inclusion and exclusion criteria, with disagreements being resolved through discussion and consensus or consultation with a third investigator. Data extracted from included studies included: (i) Basic information including title, first author, publication year, numbers of patients per group, and baseline patient characteristics; (ii) interventional measures and treatment courses for the treatment and control groups; (iii) outcome indicators; and (iv) elements necessary for risk of bias assessments. | 4 |
|  | 10b | Study screening was independently performed by two investigators with reference to the above inclusion and exclusion criteria, with disagreements being resolved through discussion and consensus or consultation with a third investigator. Data extracted from included studies included: (i) Basic information including title, first author, publication year, numbers of patients per group, and baseline patient characteristics; (ii) interventional measures and treatment courses for the treatment and control groups; (iii) outcome indicators; and (iv) elements necessary for risk of bias assessments. | 4 |
| Study risk of bias assessment | 11 | The risk of bias for included studies was independently quantified by two investigators, with disagreements being resolved through discussion with a third investigator. The risk of bias was measured with the RCT bias risk assessment tool from the Cochrane Manual 5.1.0. | 4 |
| Effect measures | 12 | RevMan5.3 was used to conduct all the present meta-analyses. When continuous data were measured using the same measurement tools and units, they were analyzed based on weighted mean difference (WMD) values, whereas they were otherwise analyzed using standard mean difference (SMD) values. Dichotomous variables were analyzed using relative risk (RR) values and 95% confidence intervals (95% CIs). The chi-squared test was used to detect heterogeneity among studies, with the I2 statistic. When no significant heterogeneity was detected (P > 0.10, I2 < 50%), results were analyzed with a fixed-effects model. When significant heterogeneity was detected (P > 0.10, I2 ≥ 50%), subgroup or sensitivity analyses were used to explore potential sources of heterogeneity. When clear sources of clinical or methodological heterogeneity had been removed, pooled meta-analyses were conducted using a random-effects model. The influence of individual studies on pooled results was assessed through sensitivity analyses. For primary outcome indicators, when 10 or more studies were available, publication bias was detected via visual inspection of funnel plots and through Egger’s test and Begg’s test. | 4 |
| Synthesis methods | 13a | RevMan5.3 was used to conduct all the present meta-analyses. When continuous data were measured using the same measurement tools and units, they were analyzed based on weighted mean difference (WMD) values, whereas they were otherwise analyzed using standard mean difference (SMD) values. Dichotomous variables were analyzed using relative risk (RR) values and 95% confidence intervals (95% CIs). The chi-squared test was used to detect heterogeneity among studies, with the I2 statistic. When no significant heterogeneity was detected (P > 0.10, I2 < 50%), results were analyzed with a fixed-effects model. When significant heterogeneity was detected (P > 0.10, I2 ≥ 50%), subgroup or sensitivity analyses were used to explore potential sources of heterogeneity. When clear sources of clinical or methodological heterogeneity had been removed, pooled meta-analyses were conducted using a random-effects model. The influence of individual studies on pooled results was assessed through sensitivity analyses. For primary outcome indicators, when 10 or more studies were available, publication bias was detected via visual inspection of funnel plots and through Egger’s test and Begg’s test. | 4 |
|  | 13b | RevMan5.3 was used to conduct all the present meta-analyses. When continuous data were measured using the same measurement tools and units, they were analyzed based on weighted mean difference (WMD) values, whereas they were otherwise analyzed using standard mean difference (SMD) values. Dichotomous variables were analyzed using relative risk (RR) values and 95% confidence intervals (95% CIs). The chi-squared test was used to detect heterogeneity among studies, with the I2 statistic. When no significant heterogeneity was detected (P > 0.10, I2 < 50%), results were analyzed with a fixed-effects model. When significant heterogeneity was detected (P > 0.10, I2 ≥ 50%), subgroup or sensitivity analyses were used to explore potential sources of heterogeneity. When clear sources of clinical or methodological heterogeneity had been removed, pooled meta-analyses were conducted using a random-effects model. The influence of individual studies on pooled results was assessed through sensitivity analyses. For primary outcome indicators, when 10 or more studies were available, publication bias was detected via visual inspection of funnel plots and through Egger’s test and Begg’s test. | 4 |
|  | 13c | RevMan5.3 was used to conduct all the present meta-analyses. When continuous data were measured using the same measurement tools and units, they were analyzed based on weighted mean difference (WMD) values, whereas they were otherwise analyzed using standard mean difference (SMD) values. Dichotomous variables were analyzed using relative risk (RR) values and 95% confidence intervals (95% CIs). The chi-squared test was used to detect heterogeneity among studies, with the I2 statistic. When no significant heterogeneity was detected (P > 0.10, I2 < 50%), results were analyzed with a fixed-effects model. When significant heterogeneity was detected (P > 0.10, I2 ≥ 50%), subgroup or sensitivity analyses were used to explore potential sources of heterogeneity. When clear sources of clinical or methodological heterogeneity had been removed, pooled meta-analyses were conducted using a random-effects model. The influence of individual studies on pooled results was assessed through sensitivity analyses. For primary outcome indicators, when 10 or more studies were available, publication bias was detected via visual inspection of funnel plots and through Egger’s test and Begg’s test. | 4 |
|  | 13d | RevMan5.3 was used to conduct all the present meta-analyses. When continuous data were measured using the same measurement tools and units, they were analyzed based on weighted mean difference (WMD) values, whereas they were otherwise analyzed using standard mean difference (SMD) values. Dichotomous variables were analyzed using relative risk (RR) values and 95% confidence intervals (95% CIs). The chi-squared test was used to detect heterogeneity among studies, with the I2 statistic. When no significant heterogeneity was detected (P > 0.10, I2 < 50%), results were analyzed with a fixed-effects model. When significant heterogeneity was detected (P > 0.10, I2 ≥ 50%), subgroup or sensitivity analyses were used to explore potential sources of heterogeneity. When clear sources of clinical or methodological heterogeneity had been removed, pooled meta-analyses were conducted using a random-effects model. The influence of individual studies on pooled results was assessed through sensitivity analyses. For primary outcome indicators, when 10 or more studies were available, publication bias was detected via visual inspection of funnel plots and through Egger’s test and Begg’s test. | 4 |
|  | 13e | RevMan5.3 was used to conduct all the present meta-analyses. When continuous data were measured using the same measurement tools and units, they were analyzed based on weighted mean difference (WMD) values, whereas they were otherwise analyzed using standard mean difference (SMD) values. Dichotomous variables were analyzed using relative risk (RR) values and 95% confidence intervals (95% CIs). The chi-squared test was used to detect heterogeneity among studies, with the I2 statistic. When no significant heterogeneity was detected (P > 0.10, I2 < 50%), results were analyzed with a fixed-effects model. When significant heterogeneity was detected (P > 0.10, I2 ≥ 50%), subgroup or sensitivity analyses were used to explore potential sources of heterogeneity. When clear sources of clinical or methodological heterogeneity had been removed, pooled meta-analyses were conducted using a random-effects model. The influence of individual studies on pooled results was assessed through sensitivity analyses. For primary outcome indicators, when 10 or more studies were available, publication bias was detected via visual inspection of funnel plots and through Egger’s test and Begg’s test. | 4 |
|  | 13f | RevMan5.3 was used to conduct all the present meta-analyses. When continuous data were measured using the same measurement tools and units, they were analyzed based on weighted mean difference (WMD) values, whereas they were otherwise analyzed using standard mean difference (SMD) values. Dichotomous variables were analyzed using relative risk (RR) values and 95% confidence intervals (95% CIs). The chi-squared test was used to detect heterogeneity among studies, with the I2 statistic. When no significant heterogeneity was detected (P > 0.10, I2 < 50%), results were analyzed with a fixed-effects model. When significant heterogeneity was detected (P > 0.10, I2 ≥ 50%), subgroup or sensitivity analyses were used to explore potential sources of heterogeneity. When clear sources of clinical or methodological heterogeneity had been removed, pooled meta-analyses were conducted using a random-effects model. The influence of individual studies on pooled results was assessed through sensitivity analyses. For primary outcome indicators, when 10 or more studies were available, publication bias was detected via visual inspection of funnel plots and through Egger’s test and Begg’s test. | 4 |
| Reporting bias assessment | 14 | RevMan5.3 was used to conduct all the present meta-analyses. When continuous data were measured using the same measurement tools and units, they were analyzed based on weighted mean difference (WMD) values, whereas they were otherwise analyzed using standard mean difference (SMD) values. Dichotomous variables were analyzed using relative risk (RR) values and 95% confidence intervals (95% CIs). The chi-squared test was used to detect heterogeneity among studies, with the I2 statistic. When no significant heterogeneity was detected (P > 0.10, I2 < 50%), results were analyzed with a fixed-effects model. When significant heterogeneity was detected (P > 0.10, I2 ≥ 50%), subgroup or sensitivity analyses were used to explore potential sources of heterogeneity. When clear sources of clinical or methodological heterogeneity had been removed, pooled meta-analyses were conducted using a random-effects model. The influence of individual studies on pooled results was assessed through sensitivity analyses. For primary outcome indicators, when 10 or more studies were available, publication bias was detected via visual inspection of funnel plots and through Egger’s test and Begg’s test. | 4 |
| Certainty assessment | 15 | RevMan5.3 was used to conduct all the present meta-analyses. When continuous data were measured using the same measurement tools and units, they were analyzed based on weighted mean difference (WMD) values, whereas they were otherwise analyzed using standard mean difference (SMD) values. Dichotomous variables were analyzed using relative risk (RR) values and 95% confidence intervals (95% CIs). The chi-squared test was used to detect heterogeneity among studies, with the I2 statistic. When no significant heterogeneity was detected (P > 0.10, I2 < 50%), results were analyzed with a fixed-effects model. When significant heterogeneity was detected (P > 0.10, I2 ≥ 50%), subgroup or sensitivity analyses were used to explore potential sources of heterogeneity. When clear sources of clinical or methodological heterogeneity had been removed, pooled meta-analyses were conducted using a random-effects model. The influence of individual studies on pooled results was assessed through sensitivity analyses. For primary outcome indicators, when 10 or more studies were available, publication bias was detected via visual inspection of funnel plots and through Egger’s test and Begg’s test. | 4 |
| **RESULTS** | | |  |
| Study selection | 16a | 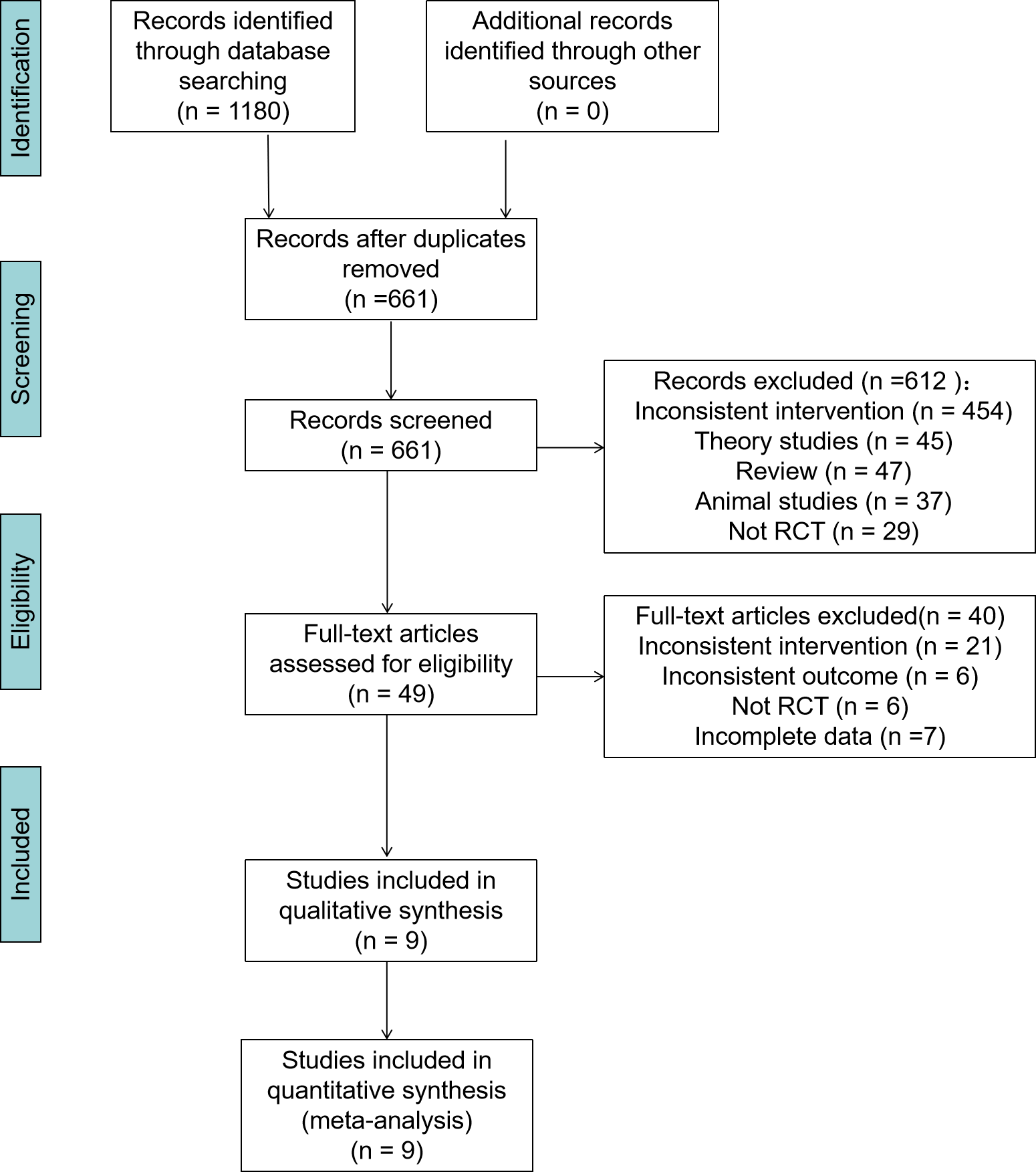 | 5 |
|  | 16b | In total, the initial search strategy retrieved 1180 potentially relevant studies, of which 661 remained following the removal of duplicates. Of these, 612 were excluded following preliminary abstract and title review, while 40 were excluded following full-text review. The remaining 9 studies were included in the final analyses. The overall screening process is detailed in Figure 1. | 4 |
| Study characteristics | 17 | \| Study cohort \| No (T/C) \| Gender \| \| Age \| \| Course (Day) \| Outcome \| \| --- \| --- \| --- \| --- \| --- \| --- \| --- \| --- \| \| T \| C \| T \| C \| \| Chen Z 2016 \| 81/78 \| 40/41 \| 40/38 \| 43.65±8.01 \| 43.93±10.56 \| 42 \| ①②⑤ \| \| Deng YH 2017 \| 59/57 \| 26/33 \| 30/27 \| 43.81±10.23 \| 44.53±11.65 \| 28 \| ①②⑥ \| \| Ding GA 2013 \| 37/38 \| - \| - \| 29.46±6.82 \| 27.62±4.24 \| 56 \| ①②③ \| \| Li P 2014 \| 100/50 \| - \| - \| - \| - \| 56 \| ①⑥ \| \| Li ZC 2016 \| 32/32 \| 15/17 \| 14/18 \| 41.6±7.8 \| 42.1±7.2 \| 42 \| ①②⑥ \| \| Li X 2017 \| 30/30 \| 22/8 \| 19/11 \| 43-79 \| 41-80 \| 28 \| ①⑥ \| \| Wang XJ 2005 \| 32/31 \| 18/14 \| 20/11 \| 19-57 \| 20-56 \| 56 \| ①② \| \| Wang ZC 2010 \| 30/30 \| 16/14 \| 15/15 \| 18-48 \| 20-51 \| 42 \| ①②⑥ \| \| Zhu W 2015 \| 28/28 \| - \| - \| - \| - \| 42 \| ①②④ \| | 5 |
| Risk of bias in studies | 18 | 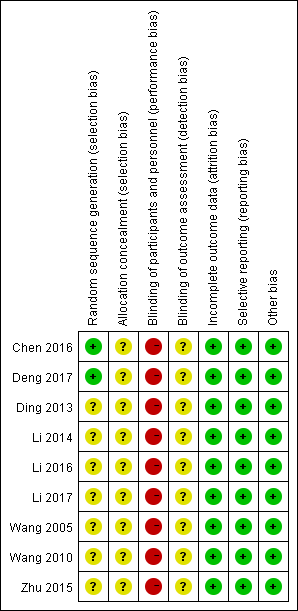 | 6 |
| Results of individual studies | 19 | 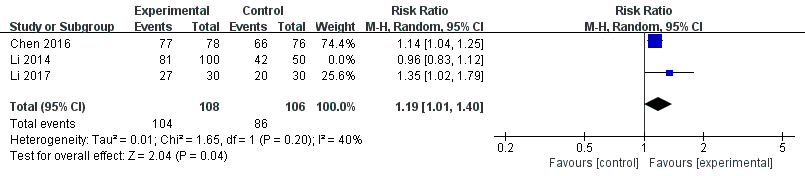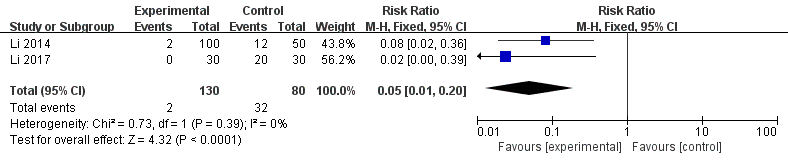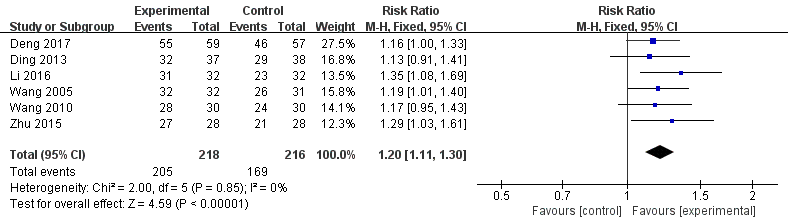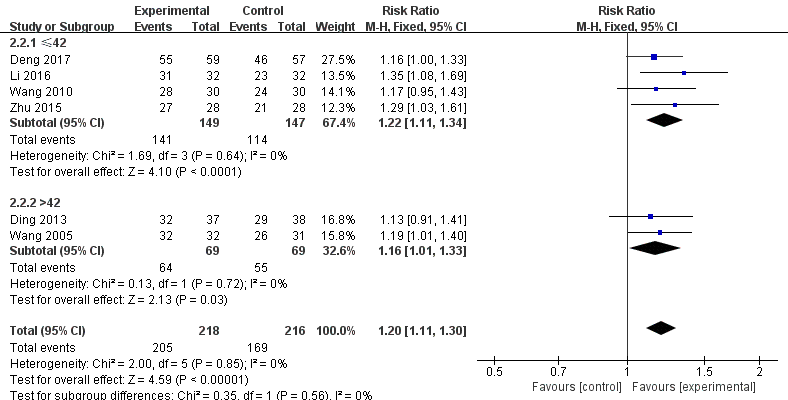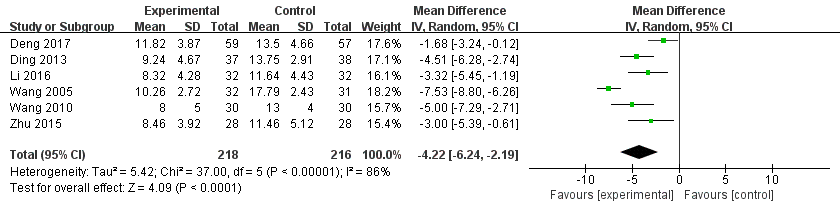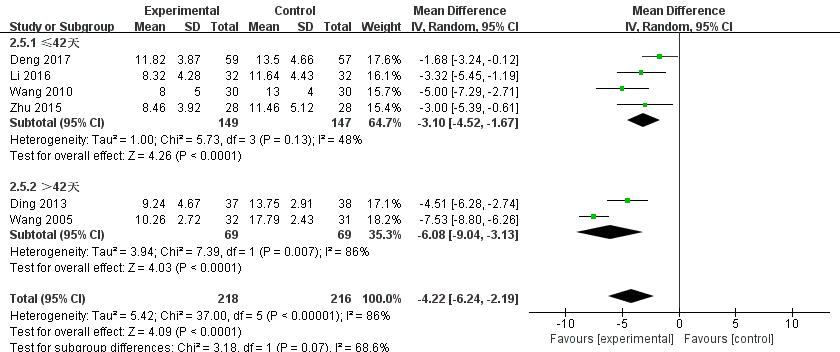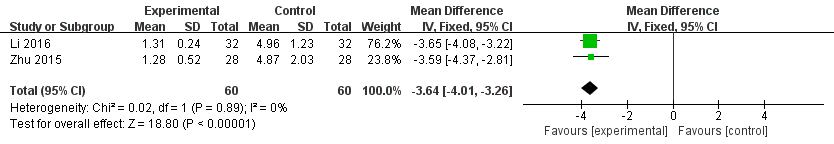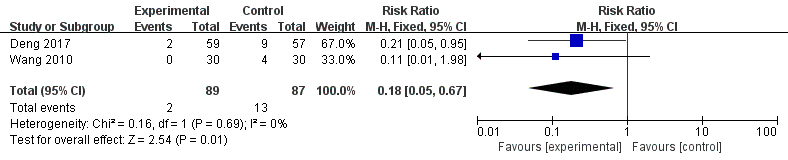 |  |
| Results of syntheses | 20a | 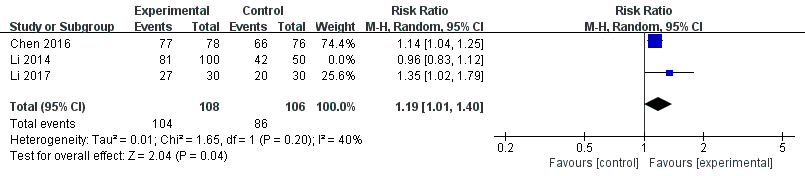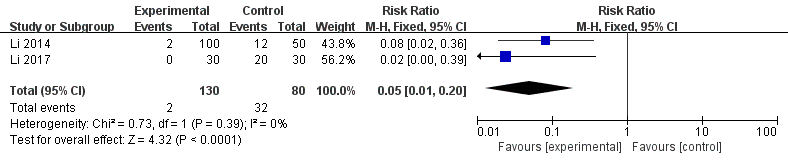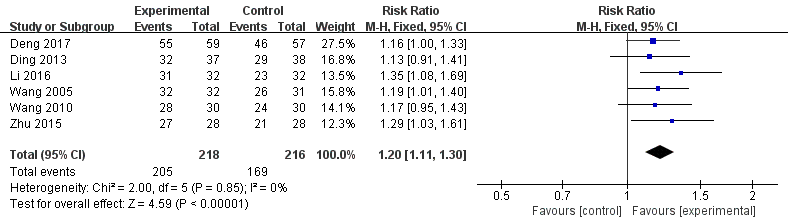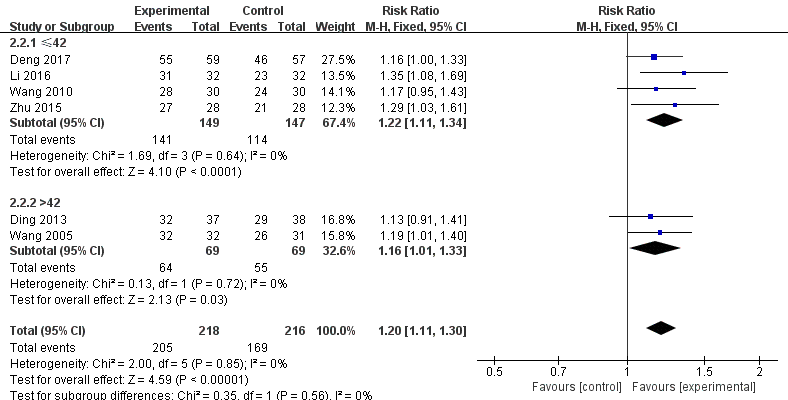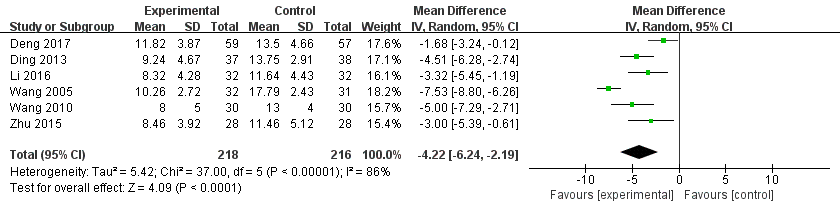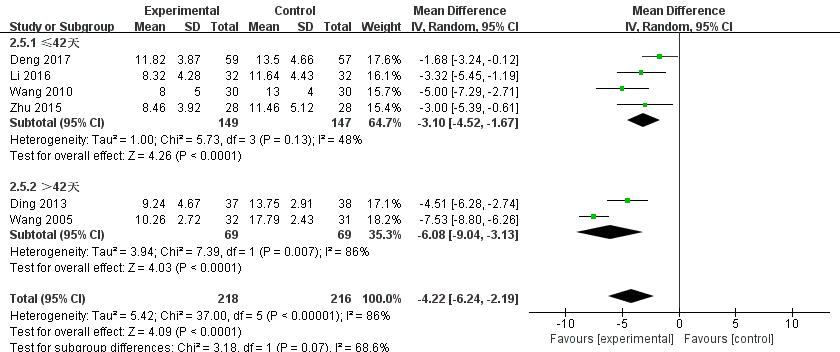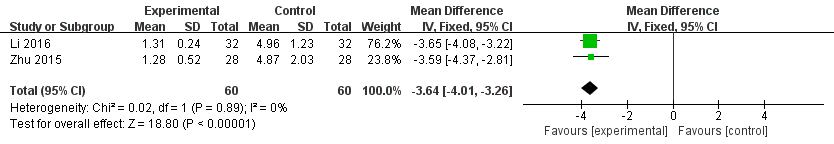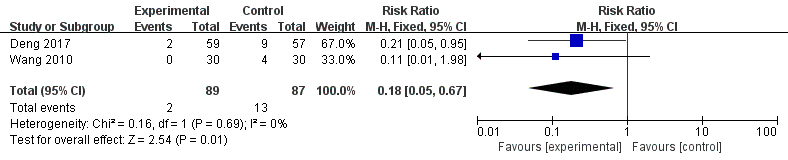 |  |
|  | 20b | 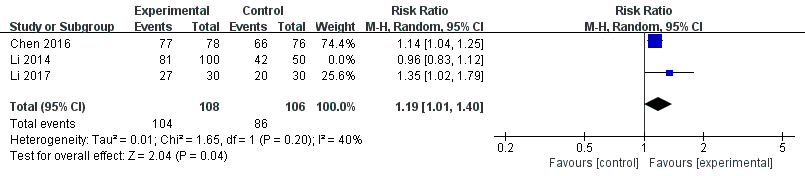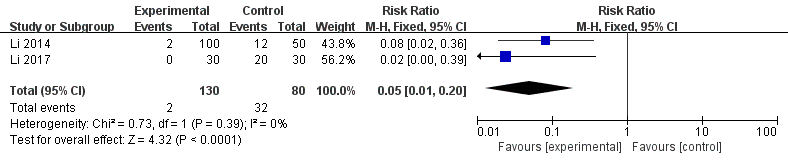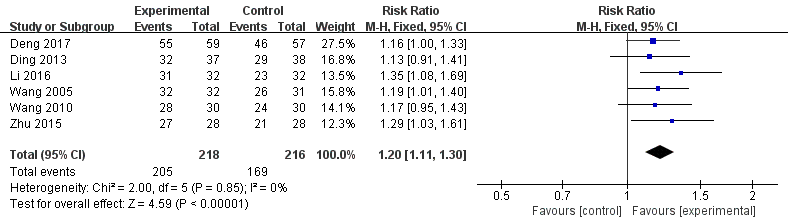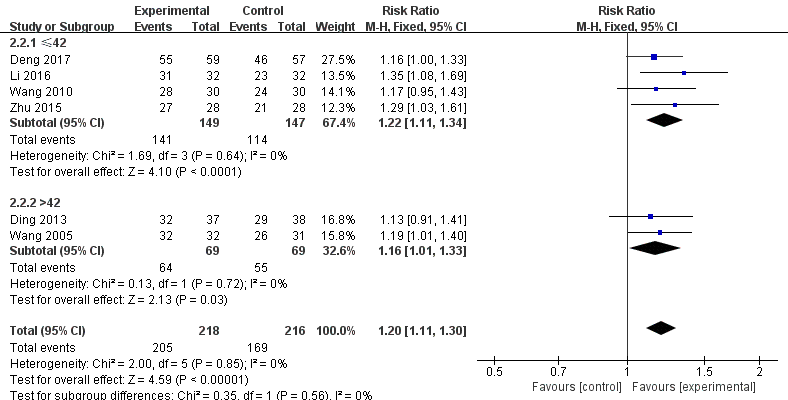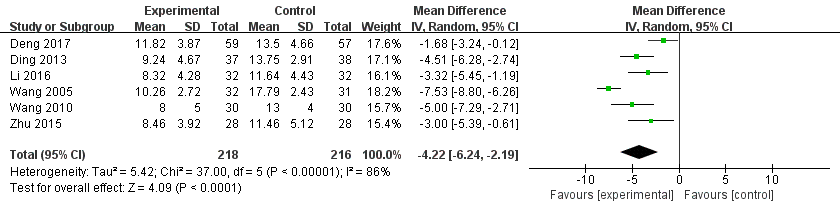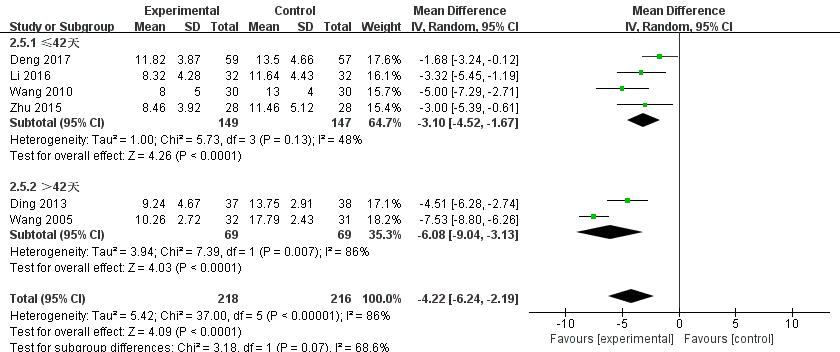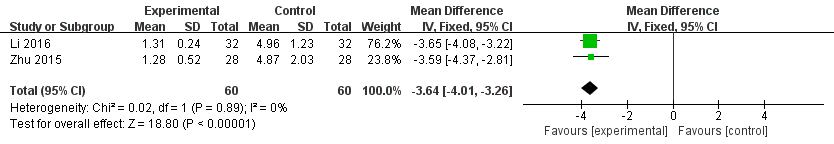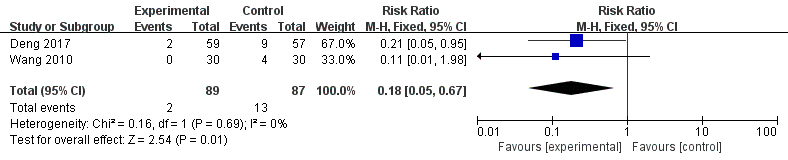 |  |
|  | 20c | 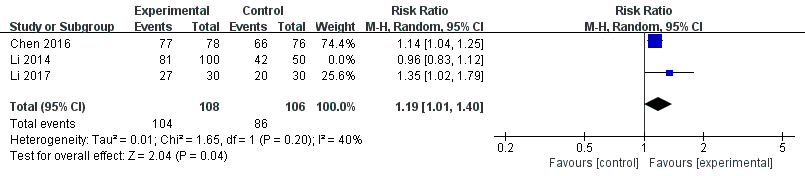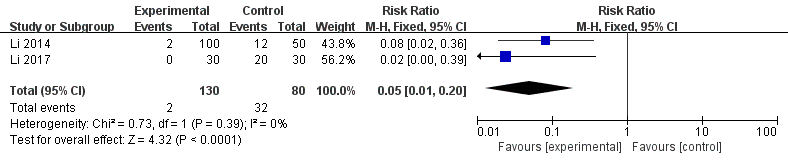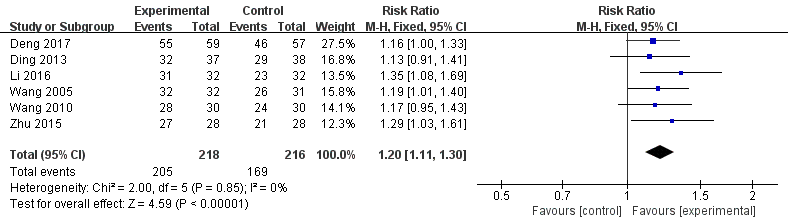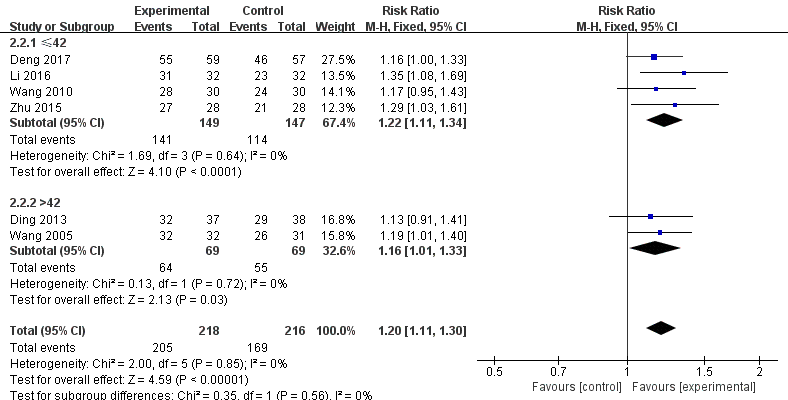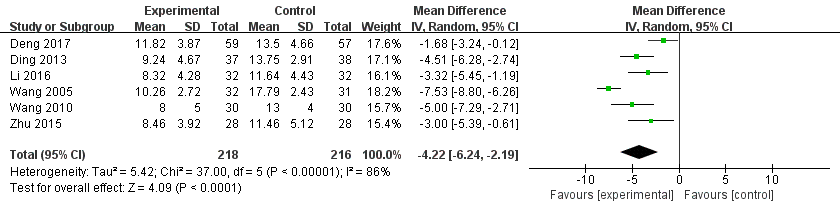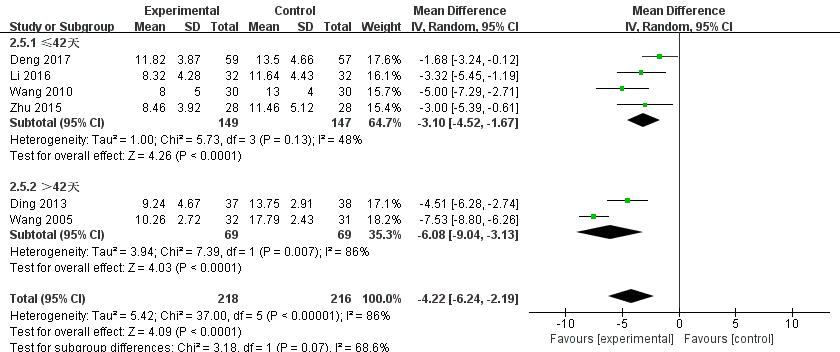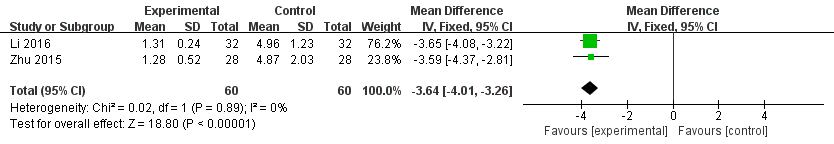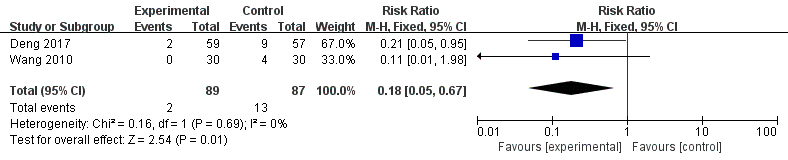 |  |
|  | 20d | 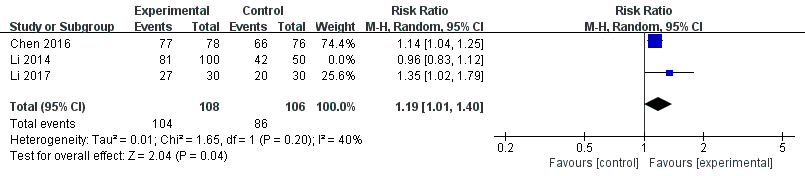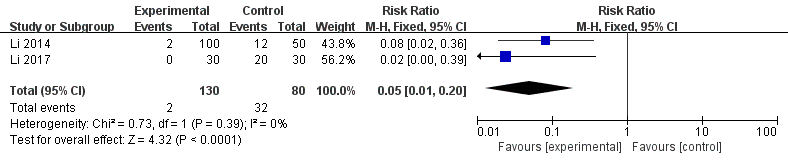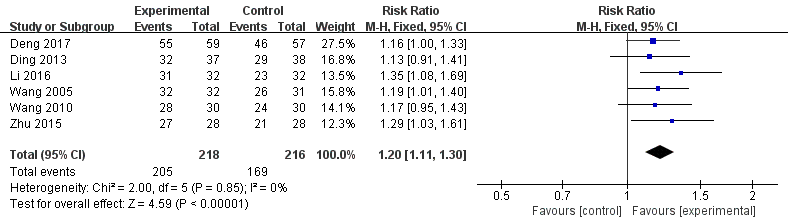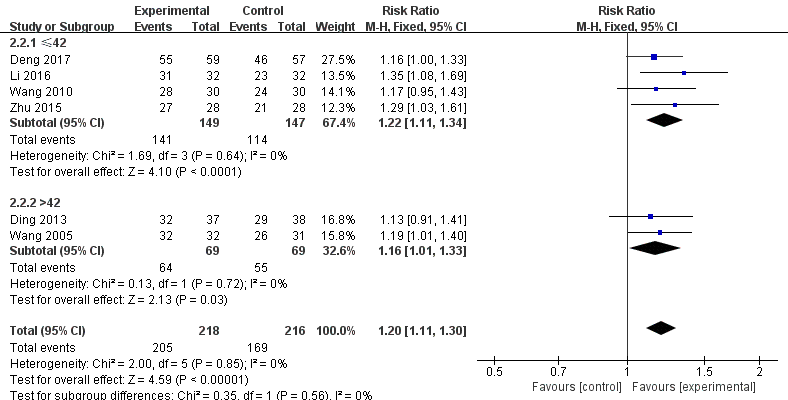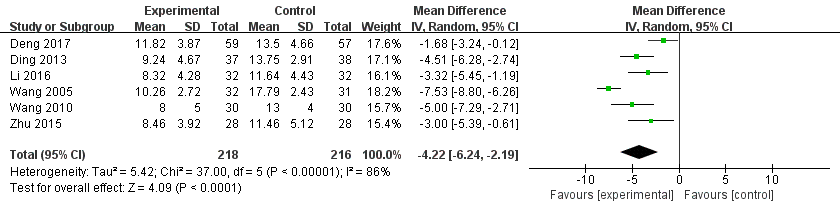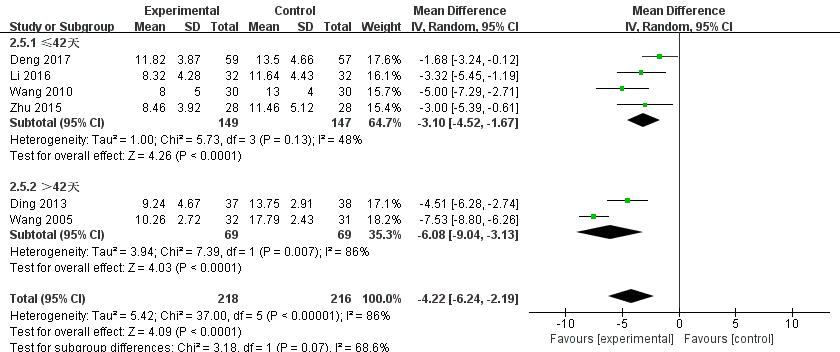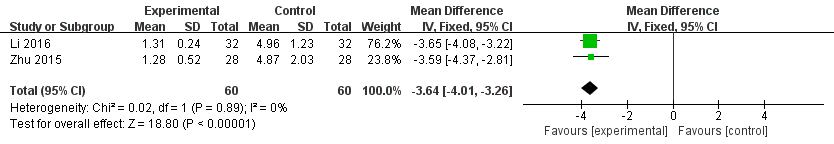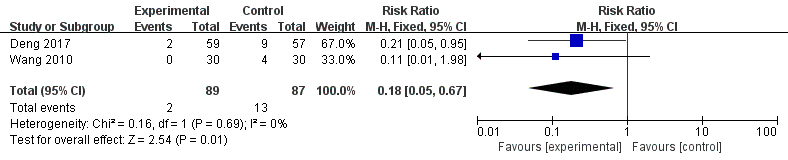 |  |
| Reporting biases | 21 | 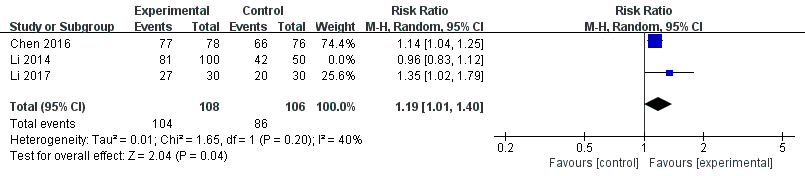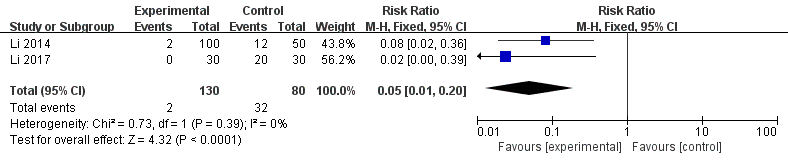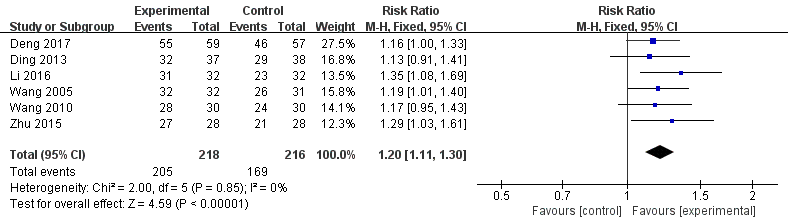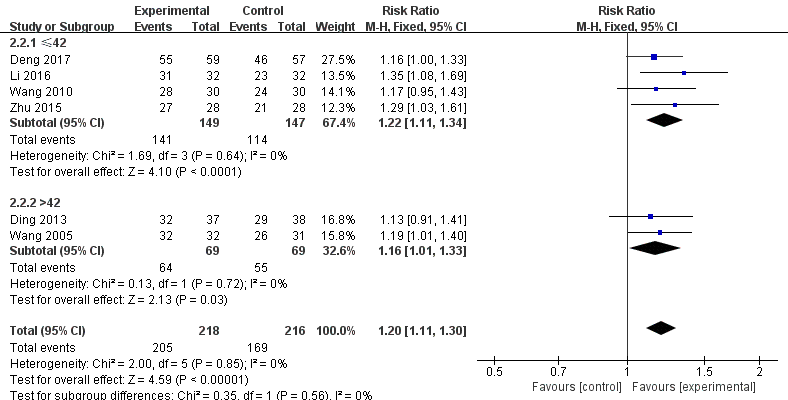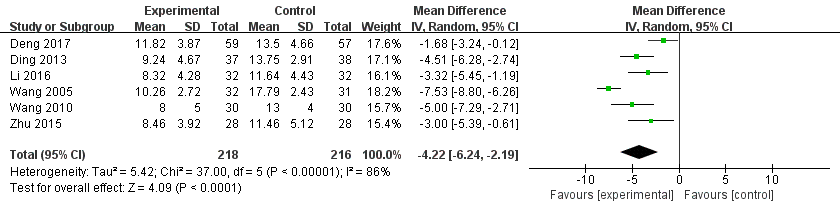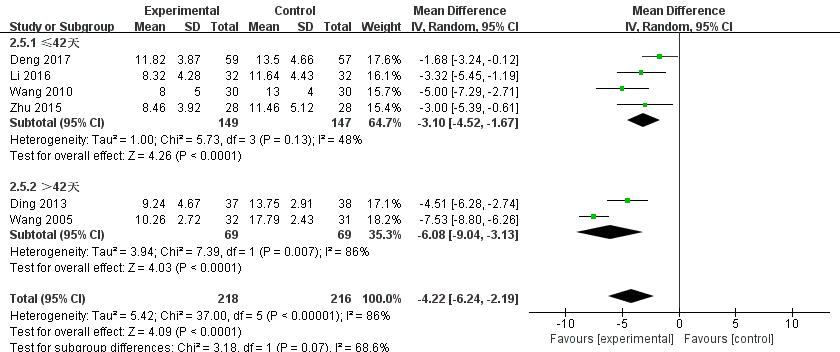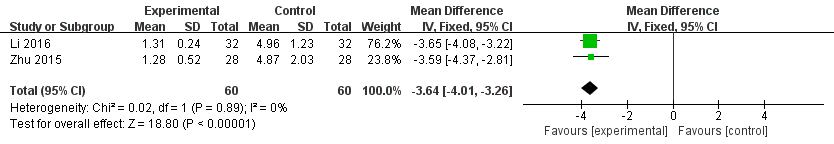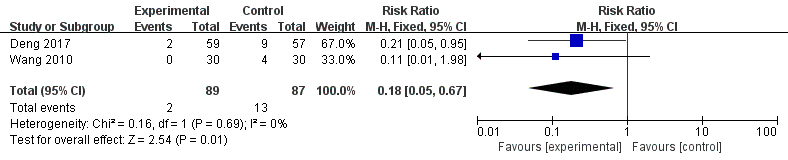 |  |
| Certainty of evidence | 22 | 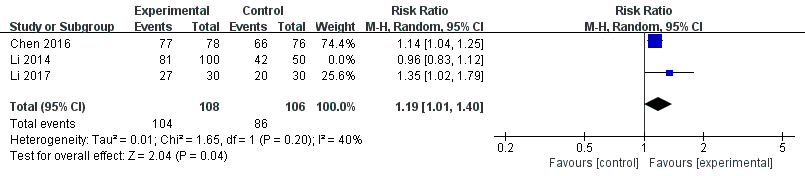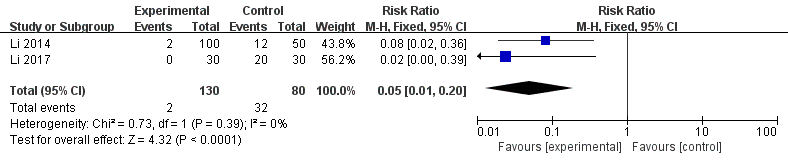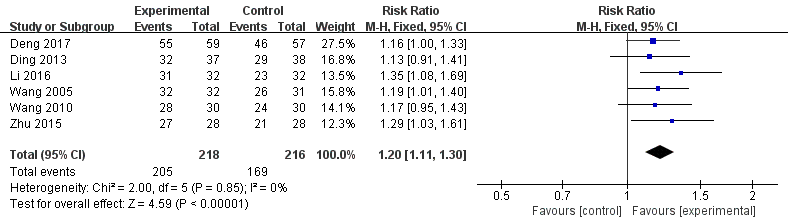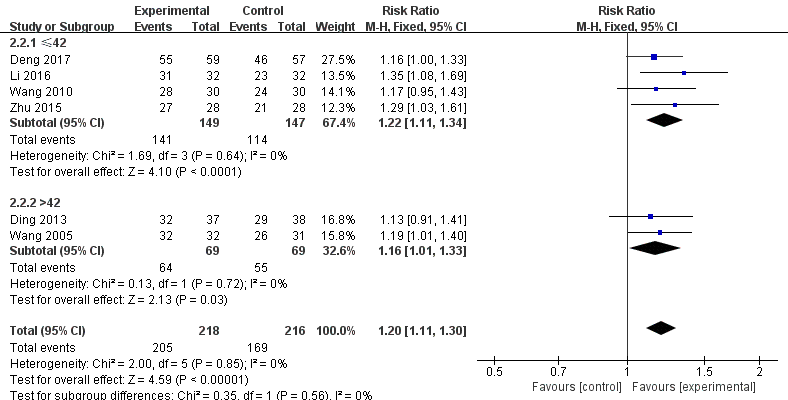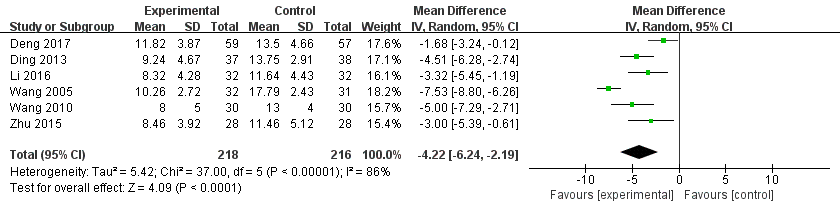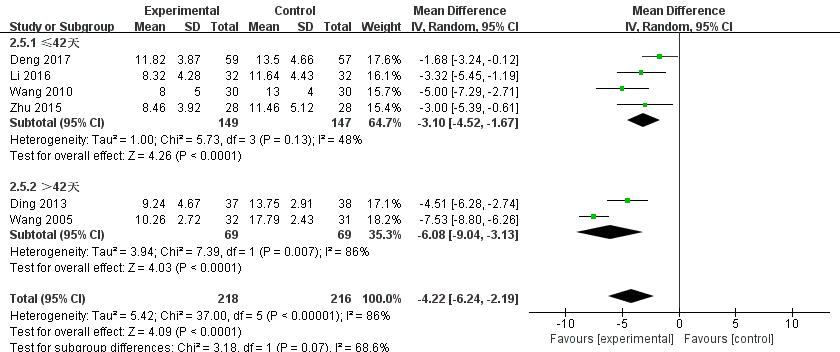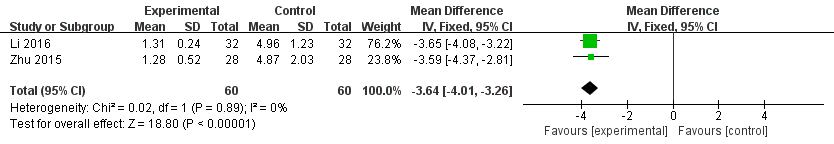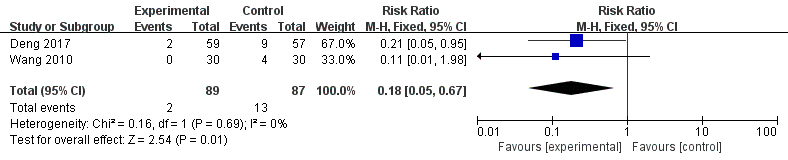 |  |
| **DISCUSSION** | | |  |
| Discussion | 23a | Here, we conducted pooled meta-analyses of 9 RCTs in which XYS was employed for the treatment of AD. The results of these analyses indicated that XYS treatment, either alone or in combination with anxiolytic agents, was superior to anxiolytic treatment alone with respect to total efficacy rates. Subgroup analyses further indicated that this effect remained evident irrespective of treatment duration. Moreover, we found that HAM-A scores, which are commonly used to assess anxiety symptoms, improved more significantly for patients treated with XYS relative to those for patients treated with anxiolytics irrespective of treatment duration or pre-treatment HAM-A scores. SAS scores are used to assess anxiety severity, and while only one study assessed the scores in patients undergoing oral XYS + anxiolytic treatment, descriptive analyses indicated that such treatment was superior to anxiolytic treatment alone. The TCM Syndrome Scale is used to evaluate patient discomfort symptoms. As relatively few studies included this scale, descriptive analyses were instead conducted, revealing that XYS treatment alone was superior to anxiolytic treatment. With respect to adverse event rates, fewer adverse reactions were reported in the XYS and XYS + anxiolytic groups relative to anxiolytic treatment alone. Meta-analyses of the results pertaining to the effects of oral XYS + anxiolytic treatment to anxiolytic treatment, scores were significantly lower in the treatment group relative to the control group. These data suggest that oral XYS is thus safe and effective as a treatment for AD, reducing drug treatment-related adverse reactions. | 11 |
|  | 23b | Anxiety is a psychological condition in which individuals experience episodes of distress and unease that can adversely affect their social function . The prolonged alertness experienced by those with anxiety can increase the risk of cardiovascular disease and cerebrovascular disease. Chinese medicinal approaches draw from thousands of years of experience, offering many advantages as treatments for psychological disorders. | 11 |
|  | 23c | This study is subject to several limitations. For one, many of the included studies did not specify the allocation concealment or blinding approaches employed, and the results may thus be susceptible to measurement bias and selection basis. In addition, all studies were from China and may thus not be generalizable. There were also differences in the dosage and composition of XYS used in these different studies, potentially influencing pooled analysis results. | 12 |
|  | 23d | Current research suggests that XYS treatment can effectively alleviate AD patient anxiety symptoms while reducing rates of adverse drug reactions as compared to anxiolytic treatment. The overall efficacy of XYS alone or in combination with anxiolytic agents was no less than that of anxiolytic agents alone in our pooled analyses. However, to validate these results, additional large scale multi-center high-quality clinical trials will be essential, thereby providing a foundation for future patient treatment. | 12 |
| **OTHER INFORMATION** | | |  |
| Registration and protocol | 24a | CRD42021285024 | 2 |
|  | 24b | PROSPERO | 2 |
|  | 24c | CRD42021285024 | 2 |
| Support | 25 | This work was supported by Tianjin Science and Technology Plan Project Science and Technology Major Project and Engineering Chronic Disease Prevention and Control Science and Technology Major Project (grant number 17ZXMFSY00100), Capability Enhancement Project for Key Disciplines of Traditional Chinese Medicine ((grant number 2018ZDXK006) | 13 |
| Competing interests | 26 | Jin Lin, Yue Ji, Jinhua Si, Xinju Li, Li Shen | 1 |
| Availability of data, code and other materials | 27 | all |  |

*From:*  Page MJ, McKenzie JE, Bossuyt PM, Boutron I, Hoffmann TC, Mulrow CD, et al. The PRISMA 2020 statement: an updated guideline for reporting systematic reviews. BMJ 2021;372:n71. doi: 10.1136/bmj.n71

For more information, visit: <http://www.prisma-statement.org/>
