# Supplementary material for: Evaluation of the Safety and Efficacy of Xiao Yao San as a Treatment for Anxiety: A Systematic Review and Meta-Analysis
Source: Evid Based Complement Alternat Med. 2022 Apr 6;2022:1319592. doi: 10.1155/2022/1319592 (PMC9007650; doi:10.1155/2022/1319592)
Supplement: Supplementary Materials — Appendix 1. Search strategy in PubMed database. (Supplementary Materials). Appendix 2. The PRISMA checklist. [file 1319592.f1.zip › 1319592.f1/search.docx]

1 "Anxiety"[MeSH Terms]/exp

2 “phobia*”[TI/AB] OR “panic”[TI/AB] OR “Angst”[TI/AB] OR“Nervousness”[TI/AB] OR “Hypervigilance”[TI/AB]OR “Anxiousness”[TI/AB] OR “Anxiet*”[TI/AB]OR “Castration Complex”[TI/AB]OR “Castration Complices”[TI/AB]OR "Catastrophizing*"[TI/AB]OR “Dental Fear*”[TI/AB]OR “Odontophobia*”[TI/AB]OR “Dental Phobia*”[TI/AB]OR “Exam Stress”[TI/AB]

3 1-2/OR

4"xiaoyao"[Supplementary Concept] /exp

5 "xiaoyao"[Text Word]) OR "xiao yao"[Text Word]

6 4-5/OR

7 "randomized controlled trial"[Publication Type] OR "controlled clinical trial" [Publication Type] OR "placebo"[TI/AB] OR "random*"[TI/AB] OR "clinical trial"[Publication Type] OR " trial"[TI/AB]

8 3 AND 6 AND 7
